# Impact of the Elephant Trunk on Distal Remodelling After Surgery for Acute Type I Aortic Dissection

**DOI:** 10.1093/icvts/ivag023

**Published:** 2026-01-23

**Authors:** You Kyeong Park, Hyoung Woo Chang, Kay-Hyun Park, Joon Chul Jung, Jae Hang Lee, Jun Sung Kim

**Affiliations:** Department of Thoracic and Cardiovascular Surgery, Soonchunhyang University Bucheon Hospital, Soonchunhyang University College of Medicine, Bucheon, Republic of Korea; Department of Thoracic and Cardiovascular Surgery, Seoul National University Bundang Hospital, Seoul National University College of Medicine, Seoul, Republic of Korea; Department of Thoracic and Cardiovascular Surgery, Seoul National University Bundang Hospital, Seoul National University College of Medicine, Seoul, Republic of Korea; Department of Thoracic and Cardiovascular Surgery, Seoul National University Bundang Hospital, Seoul National University College of Medicine, Seoul, Republic of Korea; Department of Thoracic and Cardiovascular Surgery, Seoul National University Bundang Hospital, Seoul National University College of Medicine, Seoul, Republic of Korea; Department of Thoracic and Cardiovascular Surgery, Seoul National University Bundang Hospital, Seoul National University College of Medicine, Seoul, Republic of Korea

**Keywords:** aortic dissection, elephant trunk, false lumen, remodelling

## Abstract

**Objectives:**

For surgical repair of acute type I aortic dissection, total arch replacement (TAR) with a frozen elephant trunk (FET) has been known to result in better long-term remodelling of a residual false lumen. This study was designed to investigate the impact of the elephant trunk by comparing long-term remodelling features among different extents and strategies of aortic replacement.

**Methods:**

We conducted a single-centre retrospective analysis of patients who underwent surgical repair for acute type I aortic dissection from January 2004 to June 2022. Patients were categorized based on the surgical strategy employed: non-TAR, conventional TAR, TAR with a classic elephant trunk (CET) and TAR-FET. The primary outcomes were positive remodelling of the residual false lumen and composite aortic events, with secondary outcomes focusing on early postoperative results.

**Results:**

A total of 327 patients were included. TAR, when combined with the insertion of an ET, whether it was stented or not, significantly promoted favourable aortic remodelling (*P* < .001). Compared with TAR-CET, the FET group tended towards faster false lumen thrombosis and regression, albeit without a significant difference in ultimate remodelling rates; 1-year and 5-year rates of proximal descending false lumen thrombosis were 85.4% (95% confidence interval [CI], 69.2-100) and 90.3% (95% CI, 75.9-100), respectively, after TAR-FET; additionally, these aforementioned rates were 65.7% (95% CI, 54.7-76.6) and 81.9% (95% CI, 71.8-91.9), respectively, after TAR-CET. No significant differences were observed in early postoperative outcomes or overall survival.

**Conclusions:**

The favourable remodelling of the residual false lumen after TAR-FET shown in this study is in line with results from previous studies. CET might be a reasonable alternative to FET according to the individual patient risk profiles and institutional logistics situation.

## Graphical abstract

**Figure:**
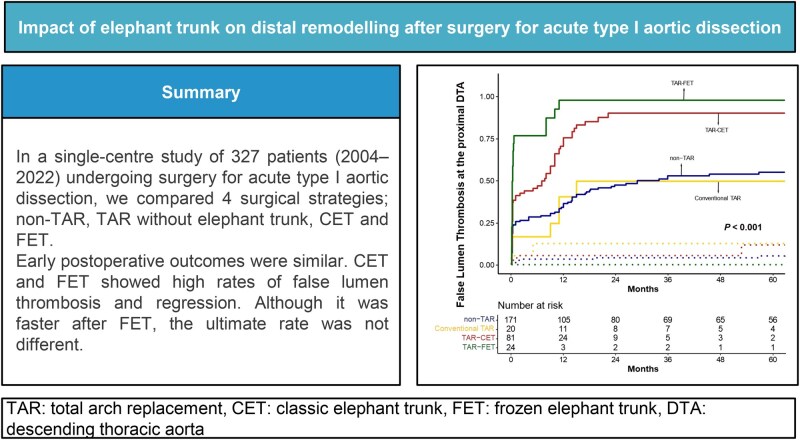

## INTRODUCTION

Acute type A aortic dissection is still perceived as a high-risk operation with considerable mortality^1^; however, experienced centres have reported remarkable outcomes, with significantly improved early survival rates and single-digit mortality.^2^^,^^3^ Despite these advances, in cases of acute type A dissection—especially De Bakey type I dissection—postoperative aneurysmal changes in the residual aorta and the need for subsequent surgical or endovascular interventions remain major concerns that impact long-term prognosis.^4^^,^^5^ Therefore, the ideal surgical strategy aims to enhance long-term outcomes without compromising early survival.

There is ongoing debate regarding whether more extensive repairs, such as total arch replacement (TAR), offer better long-term survival than does hemiarch replacement. Studies on hemiarch versus TAR have shown varied results, leaving uncertainty about the long-term survival benefits of more extensive repair.^6^ Consequently, the current guidelines recommend hemiarch replacement as the standard strategy for most patients, except for those with specific indications.^7^^,^^8^ However, over the past 2 decades, the increased adoption of TAR with the frozen elephant trunk (FET) technique has shown significantly higher rates of favourable remodelling in the residual false lumen, suggesting potential improvements in long-term prognosis.^9^^,^^10^ Notably, before the introduction of FET, studies reported favourable aortic remodelling even with TAR with a classic elephant trunk (CET), which aligns with the authors’ own experiences.^10^^,^^11^

Therefore, the aim of this study was to evaluate the potential advantages of TAR on aortic remodelling by comparing the fates of the residual false lumen among different extents of replacement and strategies, with a particular focus on whether the insertion of the FET is an essential factor for achieving favourable remodelling.

## PATIENTS AND METHODS

### Ethics statement

This study was approved by the institutional review board of Seoul National University Bundang Hospital (IRB No. B-2002–594-105) on 28 January 2020 and subsequently renewed on 4 January 2023. Individual patient consent was waived because of the retrospective nature of this study. No biological materials were collected or stored for research purposes, and no data or samples were stored in a biobank.

### Patients

This study was a single-centre, retrospective study. Consecutive patients who underwent surgical repair for acute DeBakey type I aortic dissection between January 2004 and June 2022 were included. Patients who had a completely thrombosed false lumen preoperatively (intramural haematoma) or DeBakey type II aortic dissection were excluded. The patients were divided into 4 groups on the basis of the surgical strategy employed: the non-TAR group, the conventional TAR without the elephant trunk (ET) group, the TAR with CET (TAR-CET) group and the TAR with FET (TAR-FET) group. Baseline characteristics, including demographic data, medical history, preoperative status and surgical details, were collected from medical records.

### Operative techniques

All the patients underwent emergency surgical repair. Until 2016, the right axillary artery was preferred for arterial cannulation, when our preference changed to direct ascending aortic cannulation. Femoral artery cannulation was used in a limited number of patients with unstable haemodynamics.

**Figure 1.**
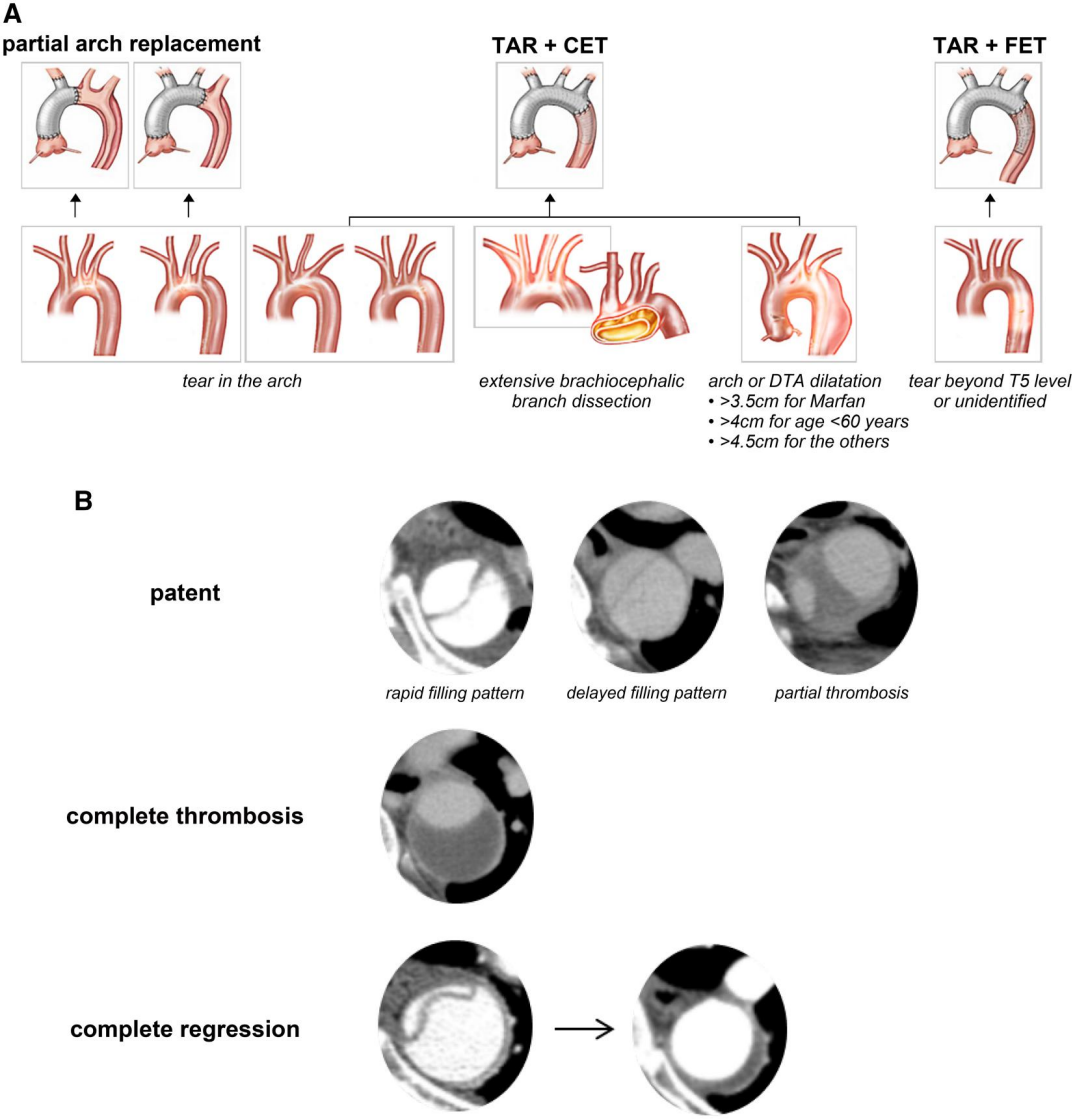
Decision About the Extent of Aortic Replacement (A) and Diagram of False Lumen Remodelling (B) Abbreviations: CET, classic elephant trunk; DTA, descending thoracic aorta; FET, frozen elephant trunk; TAR, total arch replacement.

Regardless of the extent of replacement, moderate hypothermic circulatory arrest was induced at core temperatures (bladder or rectal) between 23 °C and 27 °C. This step was followed by an aortic incision extending from the sinotubular junction to the ostium of the innominate artery to allow inspection of the aortic arch and its branches. The extent of distal repair was determined primarily by the entry tear location. Even without arch tears, TAR was selected for individuals with significant dilation of the aortic arch or the proximal descending thoracic aorta (DTA), extensive dissection involving the arch branches, known genetic conditions or younger patients (**Figure 1**).

In partial or TAR, bilateral selective cerebral perfusion was employed. With respect to TAR, false lumen obliteration at the distal anastomosis site was achieved using the adventitial inversion technique,^12^ and insertion of the ET was preferred. Commercial FET devices, such as the E-vita Open NEO hybrid stent graft system (CryoLife JOTEC GmbH, Hechingen, Germany), became available in South Korea in 2021. Prior to this, we employed an alternative technique involving the antegrade open insertion of thoracic endovascular aortic repair stent grafts. However, because this technique represents off-label use, it was reserved for select cases involving severe collapse of the true lumen in the DTA or the presence of an intimal tear in the proximal DTA. Since 2021, the choice between CET and FET has been based on the surgeon’s preference. Open insertion of a thoracic endovascular aortic repair stent graft was performed in 11 cases prior to 2021, whereas dedicated standalone FET prostheses were used in 15 cases after 2021. For the FET, device size was determined preoperatively using computed tomography (CT) and was based on the maximum true lumen diameter of the DTA at the anticipated distal landing zone—typically located at the level of the left upper pulmonary vein. Oversizing was limited to 0%-10%. In contrast, for CET, the size of the Dacron graft was determined intraoperatively by measuring the true lumen diameter at the level of the distal anastomosis using a Hegar dilator. In all patients in the TAR group, a distal anastomosis was performed in zone 3.

After completion of the distal anastomosis, cardiopulmonary bypass was resumed through arterial cannulation via a branch of the graft. The proximal anastomosis was performed while the patient was rewarmed, followed by anastomoses of the brachiocephalic branches.

### Outcome variable investigated

#### Remodelling features in postoperative CT images

Computed tomography angiography is routinely performed at the following intervals: within the first postoperative week, at 6 months, at 12 months and annually thereafter. Additional CT scans are obtained if patients develop symptoms or if aortic changes are suspected. Once complete false lumen regression is confirmed, the follow-up interval may be extended to every 2-3 years. The primary outcome was aortic remodelling of the residual false lumen at 2 different levels: The proximal DTA at the level of the main pulmonary trunk and the distal DTA at the level of the ninth thoracic vertebra. False lumen thrombosis was defined as the absence of contrast enhancement on both arterial phase and 3-minute delay phase images. False lumen regression was defined as either complete resolution of the false lumen or complete thrombosis with a residual false lumen thickness of less than 5 mm (**Figure 1**).

#### Early and long-term clinical outcomes

The secondary outcomes were early postoperative outcomes, composite aortic events and overall survival. Postoperative outcomes included 30-day mortality, in-hospital mortality, length of intubation, length of hospital stay and early postoperative complications. The long-term outcomes evaluated were all-cause mortality and composite aortic events. Composite aortic events were defined as any of the following: aortic-related death, aortic rupture, aortic dilation exceeding 10 mm compared with the preoperative diameter, a maximum aortic diameter greater than 50 mm at any level or the need for aortic intervention.

### Statistical analysis

Statistical analyses were performed using SPSS version 26 (SPSS Inc). Continuous variables are presented as means with standard deviations or medians with interquartile ranges, depending on the normality of the distribution and the presence of outliers. Categorical variables are expressed as frequencies and percentages. The χ^2^ test was used to compare the distributions of categorical variables across the 4 groups. To assess differences among groups, continuous variables were analysed using both analysis of variance for means and the Kruskal-Wallis H test for median comparisons. Kaplan-Meier survival analysis with log-rank tests was used to evaluate the rates of composite aortic events and overall survival. The cumulative incidence function with the competing risk of death and Gray’s test were used to evaluate distal false lumen remodelling. A two-tailed *P*-value less than .05 was considered statistically significant, without adjustment for multiplicity. A post hoc sensitivity analysis was performed for the composite aortic event outcome, given the limited number of events.

Additionally, the TAR-CET and TAR-FET subgroup analysis was performed using propensity score-based stabilized inverse probability of treatment weighting (IPTW), derived from a logistic regression model incorporating age, sex and primary tear location. Covariate balance was assessed using standardized mean differences and effective sample size methods. Weighted Cox proportional hazards models and IPTW-adjusted Kaplan-Meier analyses were performed, with Bonferroni-adjusted *P*-values applied.

## RESULTS

### Baseline characteristics and preoperative status

A total of 192 patients were included in the non-TAR group, 22 in the conventional TAR group, 87 in the TAR-CET group and 26 in the TAR-FET group. The baseline characteristics and preoperative status shown in **Table 1** were generally similar except for 2 aspects: The incidence of preoperative cardiopulmonary resuscitation and/or extracorporeal membrane oxygenation was greater in the non-TAR group, whereas visceral malperfusion was more common in the TAR-CET and TAR-FET groups and lower limb malperfusion was more common in the TAR-FET group. In the non-TAR group, 73.4% of tears were located in the ascending aorta/sinus of Valsalva, which was the highest percentage among all groups. Whereas 27.6% of patients in this group had a tear in the aortic arch, they were still classified as being in the non-TAR group because only the proximal part of the arch was replaced, ensuring that no patients were left with a residual arch tear. Overall, 54 patients (16.5%) had intimal tears that were unidentifiable via a median sternotomy or were located in the DTA that could not be resected, without any identifiable tear in the ascending aorta or aortic arch. Among them, the majority (36 patients, 66.7%) underwent TAR with CET or FET. Accordingly, the TAR-FET group demonstrated the highest proportion of DTA or undetermined intimal tears, with a significantly greater incidence of 58%.

**Table 1.** Baseline Characteristics and Preoperative Status

	Non-TAR (*n* = 192)	Conventional TAR (*n* = 22)	TAR-CET (*n* = 87)	TAR-FET (*n* = 26)	*P*-value
Age (years)	58 ± 15	49 ± 14	55 ± 12	59 ± 15	.02
Sex, male, *n* (%)	108 (56)	20 (91)	58 (67)	18 (69)	.008
Comorbidities, *n* (%)					
Hypertension	131 (68)	9 (41)	51 (59)	22 (85)	.006
Diabetes mellitus	17 (9)	0 (0)	2 (2)	2 (8)	.115
Hyperlipidaemia	17 (9)	0 (0)	10 (12)	6 (23)	.050
Cerebrovascular disease	15 (8)	2 (9)	2 (2)	1 (4)	.291
Chronic kidney disease	8 (4)	1 (5)	4 (5)	1 (4)	.998
Coronary artery disease	16 (8)	0 (0)	3 (4)	2 (8)	.259
Marfan syndrome	22 (12)	4 (18)	5 (6)	1 (4)	.171
Preoperative ECMO or CPR	21 (11)	2 (9)	1 (1)	1 (4)	.033
Neurological deficit	45 (24)	3 (14)	15 (17)	5 (20)	.531
Cardiac tamponade	27 (14)	3 (14)	2 (2)	5 (19)	.016
Malperfusion, *n* (%)					
Coronary	17 (9)	1 (5)	10 (12)	0 (0)	.277
Cerebral	41 (21)	3 (14)	20 (23)	7 (27)	.716
Visceral	19 (10)	1 (5)	15 (17)	7 (27)	.030
Lower limb	32 (17)	3 (14)	15 (17)	7 (27)	.588
Renal	33 (17)	2 (9)	17 (20)	4 (15)	.702
Location of intimal tear, *n* (%)					
Ascending aorta/sinus of Valsalva	141 (73)	5 (23)	37 (43)	11 (42)	<.001
Aortic arch	53 (28)	15 (68)	29 (33)	5 (19)	<.001
DTA or undetermined	14 (7)	4 (18)	29 (33)	15 (58)	<.001

The values are presented as the means with standard deviations or numbers with percentages.

Abbreviations: CET, classic elephant trunk; CPR, cardiopulmonary resuscitation; DTA, descending thoracic aorta; ECMO, extracorporeal membrane oxygenation; FET, frozen elephant trunk.

### Operative data and early postoperative outcomes

In the non-TAR group, a root procedure was performed in 31.8% of the patients, indicating a trend towards a higher frequency than in the other groups (**Table 2**). The total circulatory arrest time for low-body arrest averaged approximately 65 minutes across all TAR groups, which was approximately 20 minutes longer than for the non-TAR group. Similarly, the aortic cross-clamp time, cardiopulmonary bypass time and total operation time followed a similar pattern. Notably, compared with the FET group, the CET group did not require longer operative times.

**Table 2.** Operative Data and Early Postoperative Outcomes

	Non-TAR (*n* = 192)	Conventional TAR (*n* = 22)	TAR-CET (*n* = 87)	TAR-FET (*n* = 26)	*P*-value
Combined procedure, *n* (%)					
Root procedure	61 (32)	4 (18)	15 (17)	6 (23)	.057
Coronary artery bypass surgery	12 (6)	1 (5)	2 (2)	0 (0)	.320
Operation time, min					
Total operation time	317 ± 75	365 ± 117	323 ± 78	349 ± 80	.020
CPB time	167 ± 49	220 ± 85	183 ± 48	185 ± 60	<.001
ACC time	117 ± 46	148 ± 58	133 ± 46	139 ± 57	.002
TCA time	42 ± 15	64 ± 18	66 ± 15	65 ± 18	<.001
Early complications, *n* (%)					
Reoperation due to bleeding	36 (19)	3 (14)	14 (16)	7 (27)	.590
Stroke	14 (7)	2 (9)	4 (5)	0 (0)	.416
Permanent neurological deficit	9 (5)	1 (5)	3 (4)	0 (0)	.702
Temporary neurological deficit	5 (3)	1 (5)	1 (1)	0 (0)	.619
Paraplegia	2 (1)	1 (5)	2 (2)	0 (0)	.498
Respiratory complications	67 (35)	8 (36)	26 (30)	7 (27)	.743
Tracheostomy	14 (7)	2 (9)	6 (7)	1 (4)	.904
Vocal cord palsy	7 (4)	4 (18)	15 (17)	2 (8)	<.001
Mediastinitis	4 (2)	2 (9)	1 (2)	1 (4)	.172
Haemodialysis	27 (14)	2 (9)	14 (16)	1 (4)	.391
Time to extubation, hours	19 (12-26)	12 (9-20)	16 (10-22)	13 (8-18)	.627
Hospitalization length, days	12 (6-18)	14 (8-22)	14 (7-21)	13 (6-19)	.328
30-Day deaths, *n* (%)	20 (10)	3 (14)	6 (7)	2 (8)	.703
In-hospital deaths, *n* (%)	24 (13)	3 (14)	7 (8)	2 (8)	.645
Composite aortic event at proximal DTA	62 (32)	6 (27)	8 (9)	3 (12)	<.001
Open surgery	23 (12)	3 (14)	2 (2)	2 (8)	.062
TEVAR	1 (1)	0 (0)	0 (0)	0 (0)	.872
Aortic rupture	3 (2)	0 (0)	0 (0)	0 (0)	.546
Increase in size	57 (30)	5 (23)	8 (9)	3 (12)	<.001

The values are presented as numbers with percentages, means with standard deviations or medians with interquartile ranges.

Abbreviations: ACC, aortic cross-clamp; CET, classic elephant trunk; CPB, cardiopulmonary bypass; DTA, descending thoracic aorta; FET, frozen elephant trunk; TAR, total arch replacement; TCA, total circulatory arrest; TEVAR, thoracic endovascular aortic repair.

The incidence of vocal cord palsy was significantly lower in the non-TAR and TAR-FET groups than in the conventional TAR and TAR-CET groups (*P* < .001), and no cases of spinal cord ischaemia were observed. Other early complications did not differ significantly among the 4 groups.

### Aortic remodelling

**Figure 2.**
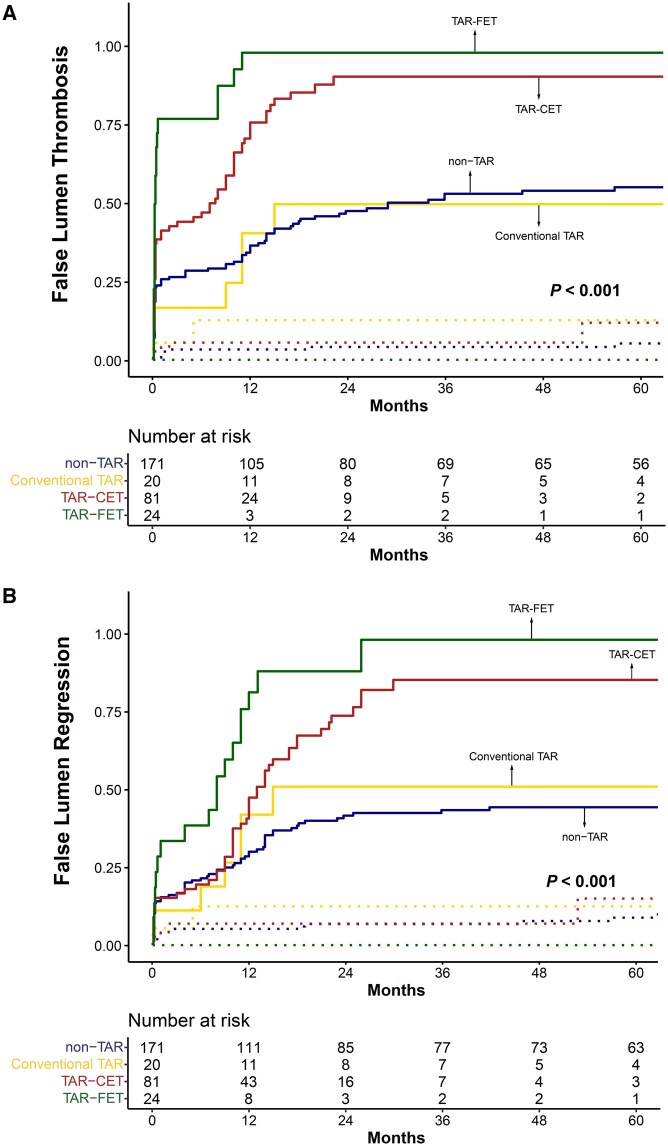
False Lumen Thrombosis (A) and False Lumen Regression (B) at the Proximal DTA Abbreviations: CET, classic elephant trunk; FET, frozen elephant trunk; TAR, total arch replacement.

**Figure 3.**
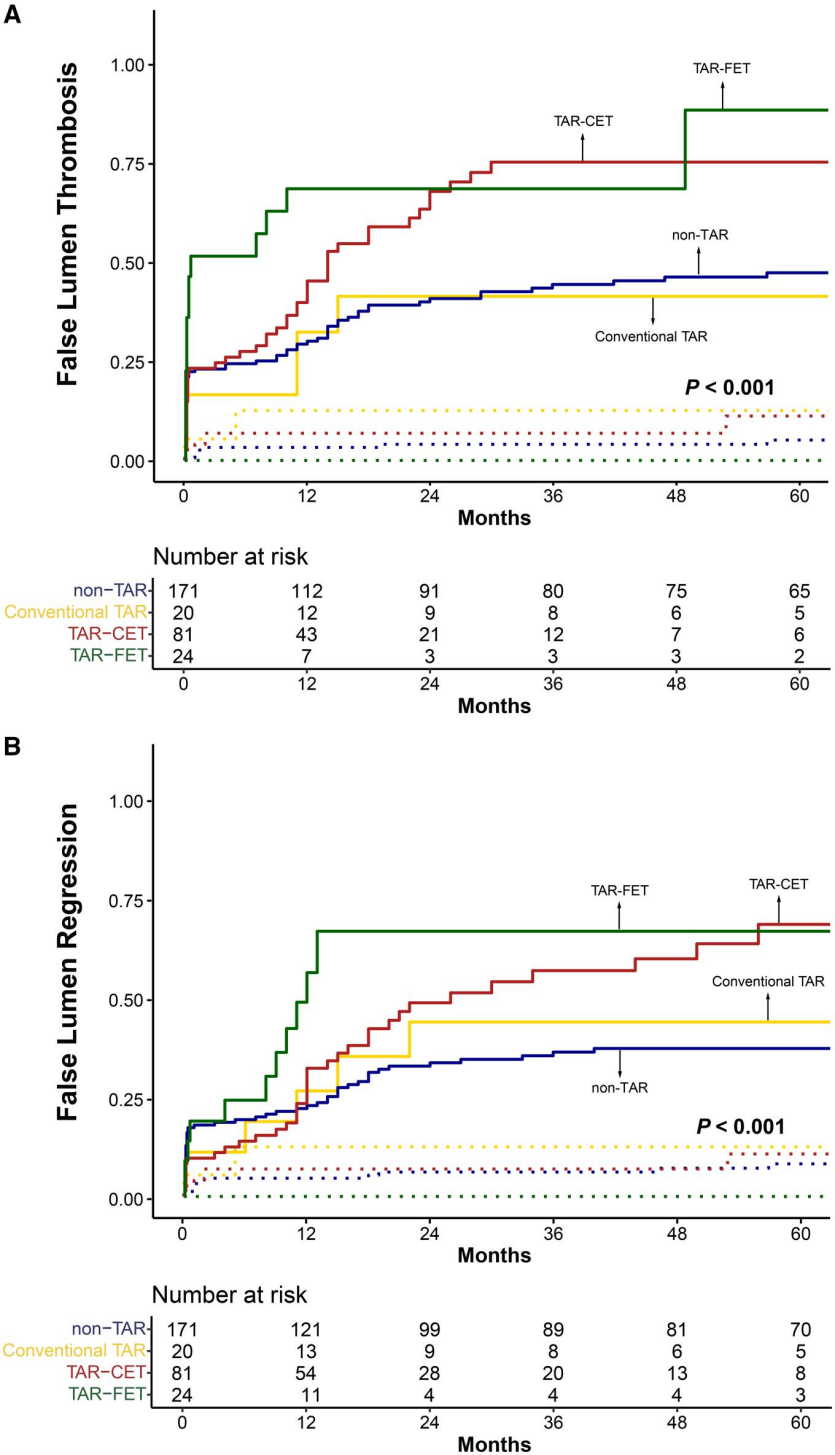
False Lumen Thrombosis (A) and False Lumen Regression (B) at the Distal DTA Abbreviations: CET, classic elephant trunk; FET, frozen elephant trunk; TAR, total arch replacement.

Among the 327 patients, 296 underwent 1 or more postoperative CT scans. The mean interval between the initial surgery and the most recent CT scan was 45.8 ± 48.3 months. The incidences of false lumen thrombosis and regression are illustrated in **Figures 2 and 3**. At the proximal DTA level, false lumen thrombosis was more frequently observed in the TAR-CET and TAR-FET groups. Specifically, in the TAR-FET group, 75% of patients demonstrated false lumen thrombosis by 8 months and 90% by 11 months. In comparison, the TAR-CET group demonstrated 75% by 14 months and 87% by 22 months. Although the rate of thrombosis progression differed, the final outcomes were similar between the groups. A similar pattern was observed for the incidence of false lumen regression. This pattern was also evident at the distal DTA level, where false lumen thrombosis and regression occurred more frequently in the TAR-CET and TAR-FET groups than in the conventional TAR or non-TAR groups. In the subgroup analysis between the TAR-CET and TAR-FET groups, stabilized IPTW yielded adequate covariate balance, with only a mild residual imbalance in age (standardized mean difference = 0.155). The IPTW-adjusted Cox proportional hazards models and weighted Kaplan-Meier analyses demonstrated no significant differences regarding proximal and distal DTA thrombosis and regression (**Figure S1**). The postoperative trends in the true lumen-to-aorta diameter ratio at the proximal and distal DTA levels are shown in **Figure S2**.

### Composite aortic events and overall survival

**Figure 4.**
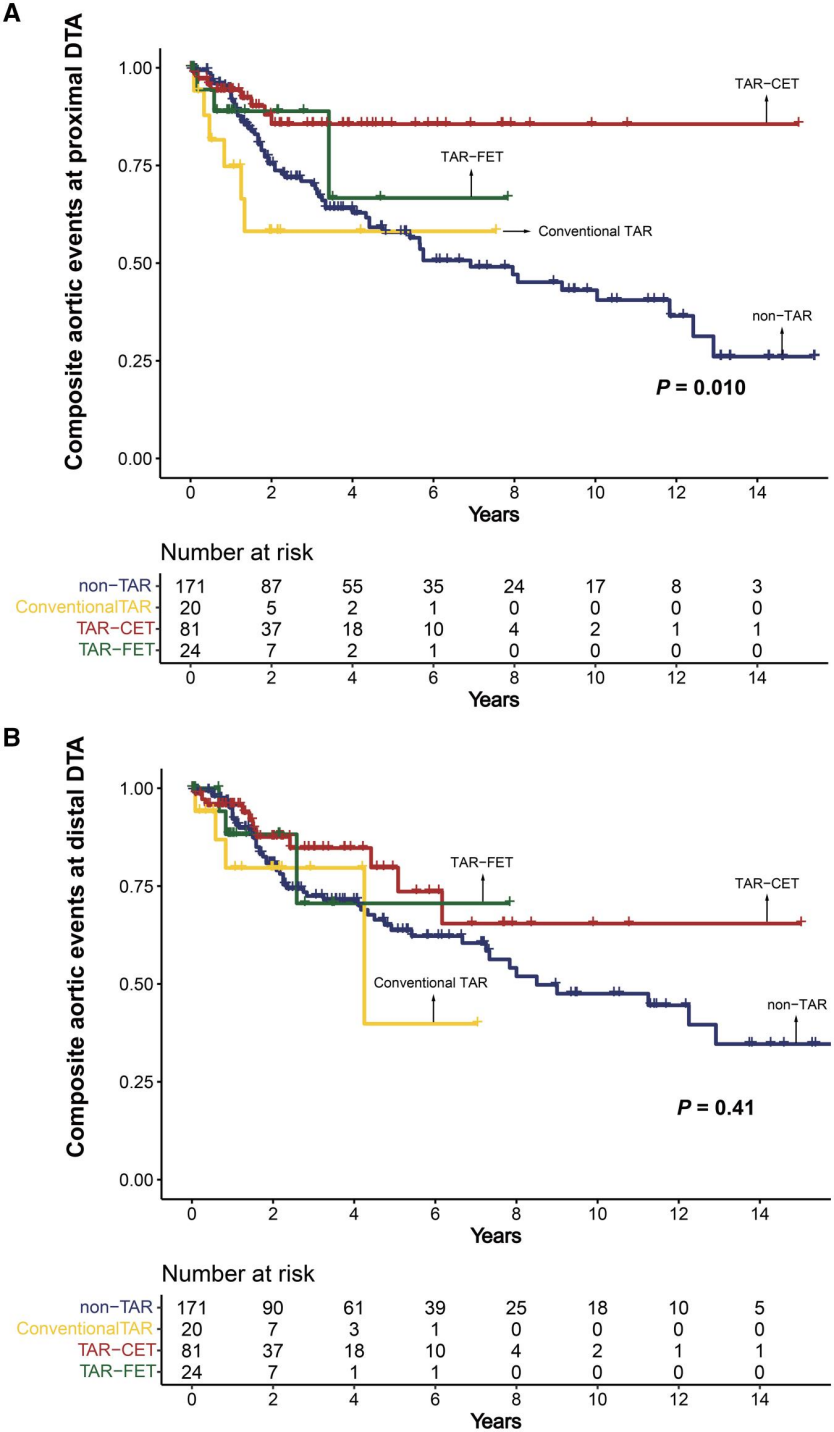
Composite Aortic Events at the Proximal DTA (A) and Distal DTA (B) Abbreviations: CET, classic elephant trunk; DTA, descending thoracic aorta; FET, frozen elephant trunk; TAR, total arch replacement.

**Figure 5.**
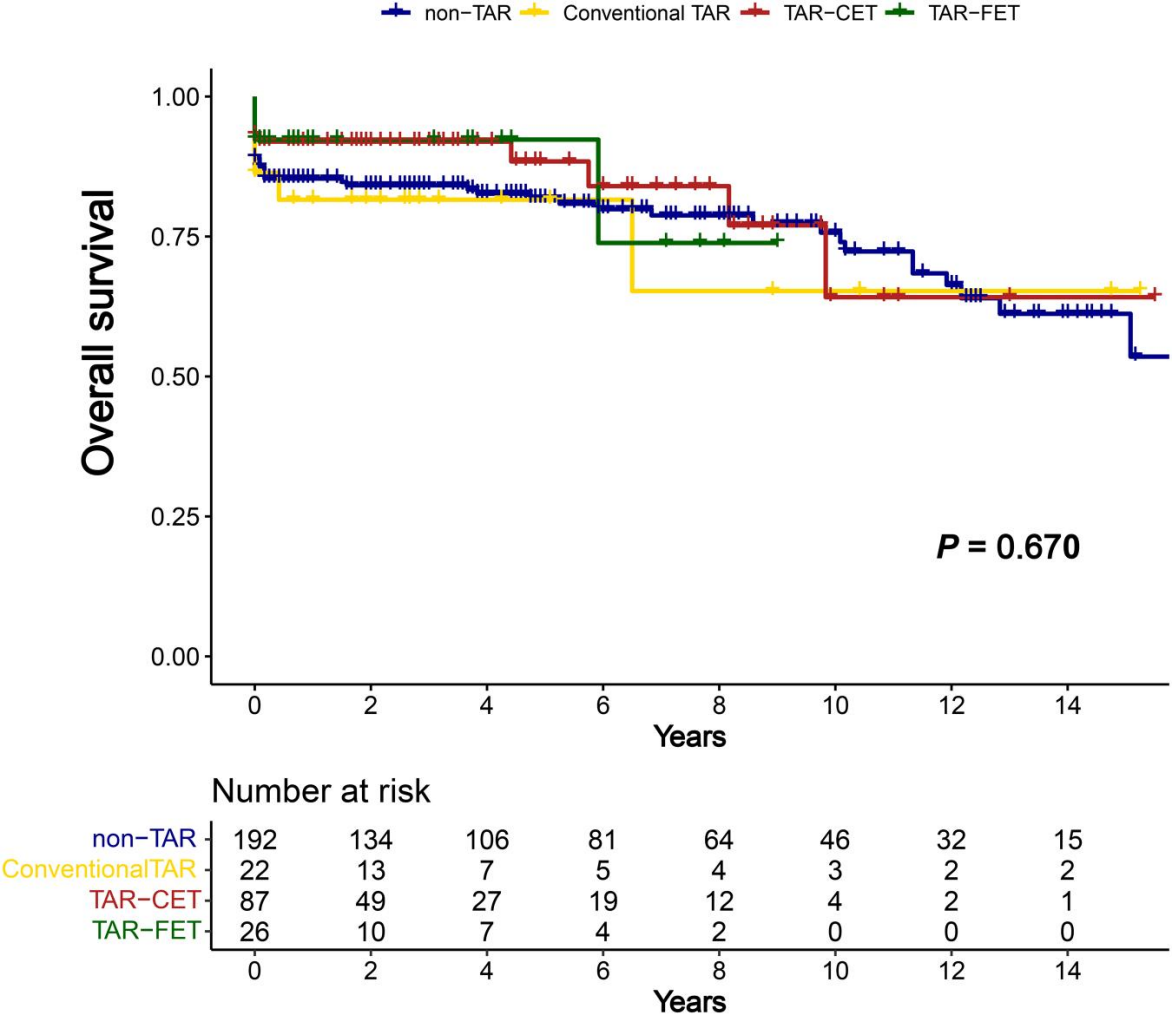
Overall Survival Abbreviations: CET, classic elephant trunk; FET, frozen elephant trunk; TAR, total arch replacement.

Composite aortic events at the proximal DTA occurred less frequently in the following order: TAR-CET, TAR-FET, conventional TAR and non-TAR (*P* = .010) (**Table 2** and **Figure 4**). Regarding the results that composite aortic events appeared to occur more frequently in the TAR-FET than in the TAR-CET group, a post hoc sensitivity analysis using the Schoenfeld approximation was performed and indicated that the minimum detectable hazard ratio with 80% power at a 2-sided α of 0.05 was approximately 7.5. Thus, this finding should be interpreted as reflecting limited statistical power. In contrast, there were no significant differences in composite aortic events at the distal DTA level and in overall survival among the 4 groups (**Figure 5**).

## DISCUSSION

This study has 2 main findings. First, the ET technique, whether performed using FET or CET, resulted in more favourable aortic remodelling than did conventional TAR or non-TAR procedures. Second, and notably, CET achieved a degree of aortic remodelling comparable to that observed with FET.

Our findings are consistent with those reported in the growing number of articles that acknowledge the efficacy of FET in managing aortic dissections. Dohle et al.^9^ analysed the residual false lumen in 70 patients treated with FET for acute aortic dissection and reported 90% positive remodelling at the stent graft level and 65% positive remodelling downstream of the coeliac trunk. Similarly, our study demonstrated that FET offers unique advantages by promoting more rapid and consistent thrombosis and regression of the false lumen, resulting in aortic stabilization and reducing the risk of further aortic dilatation and rupture.

Although numerous studies have already demonstrated favourable outcomes with FET, a noteworthy finding of this study is that the TAR-CET group also achieved favourable aortic remodelling comparable to that of the TAR-FET group. There are several possible explanations for these results. False lumen patency is maintained primarily by unresected entry tears, which can originate from the aortic arch or proximal DTA. Both CET and FET share the ability to mitigate these factors by resecting major entry tears. Additionally, by replacing the brachiocephalic branches, both techniques help prevent these vessels from serving as secondary entry tears. Moreover, the presence of a distal anastomotic new entry has been suggested as another mechanism contributing to false lumen patency.^13^ In our experience, the insertion of the ET—whether stented or not—helps to seal large needle holes in the dissection membrane that could otherwise become a distal anastomotic new entry, thereby further promoting favourable aortic remodelling.

Compared with the CET, the FET is associated with several disadvantages. The primary disadvantage of an FET is the increased risk of paraplegia. Multicentre research by Leontyev et al.^14^ revealed paraparesis and paraplegia rates of 6.5% in patients with type I acute aortic dissection. This risk may result from spinal cord ischaemia during circulatory arrest, occlusion of intercostal arteries by the stent graft, embolism, perioperative hypotension, malperfusion or a combination of these factors.^15^ Although early false lumen thrombosis is generally considered beneficial after TAR-FET, it may be detrimental in cases where the lower thoracic intercostal arteries originate entirely from the false lumen. A recent study revealed that patients with false lumen-dependent segmental arteries at the T9–L3 level had a significantly higher incidence of spinal cord injury following TAR-FET (34.3% vs 2.7%).^16^ Additionally, a distal stent graft-induced new entry (SINE) is a recognized complication following TAR-FET. Kreibich et al.^17^ reported a distal SINE incidence of 12.7% after FET, highlighting excessive stent graft oversizing and aortic remodelling as key risk factors for its development.

Despite these disadvantages associated with FET, some surgeons may still have concerns regarding the use of CET. The relatively small graft size in the CET might raise concerns about inadequate or delayed true lumen expansion. However, our data suggested that CET can still achieve favourable aortic remodelling. Similarly, one of the frequently cited advantages of FET is that it allows for a more proximalized distal anastomosis (zone 2 or even zone 1), making the procedure technically easier.^18^ However, reports have demonstrated that TAR with a proximalized anastomosis can also be performed using CET,^19^ and a zone 2 anastomosis was feasible in a more time-efficient manner, particularly when the distal anastomosis was selectively proximalized.

Whereas the incidence of distal aortic reintervention has been reduced with the use of the ET technique, reintervention remains necessary in certain cases. The effectiveness of endovascular aortic repair for post-type A residual type B dissection is still unpredictable. One study on hybrid aortic repair for chronic residual aortic dissection reported aneurysmal progression in the thoraco-abdominal aorta in 41.4% of cases and a reintervention rate of 34.5%.^20^ Therefore, unless the patient is considered high risk, open surgery remains the most definitive treatment option. The advantages of the CET technique in such scenarios have been demonstrated in quite a few studies.^21^^,^^22^ Although the FET has been shown to facilitate additional thoraco-abdominal aortic interventions,^23^ surgeons have not frequently encountered staged thoracic or thoraco-abdominal aorta repair following the FET. This situation represents a challenge that must be addressed in the near future.

In summary, whereas FET has recently become the predominant device of choice, CET may serve as a valuable alternative under certain conditions^1^: When early false lumen thrombosis may lead to paraplegia, such as in cases where the lower thoracic intercostal arteries originate entirely from the false lumen^2^; in patients with a tortuous DTA, which may increase the risk of distal SINE; and in countries where the use of FET is limited because of its high cost.

Our study has several limitations. First, it is limited by the nature of a single-centre retrospective design. This study spans more than 2 decades of operating experience; therefore, the possibility of a learning curve cannot be excluded. Second, the recent introduction of a commercial FET product into our country led to a smaller cohort of patients and shorter follow-up periods. Since the beginning of this study period, we have performed FETs more frequently, and these patients have demonstrated a similar rate of positive remodelling. We anticipate that further follow-up with a larger cohort will allow us to provide more robust data, including the long-term benefit of long-term survival and aortic events at the distal thoracic and abdominal aorta, which we could not demonstrate in this study. Third, preoperative anticoagulant or antiplatelet medication could not be reliably analysed because a substantial proportion of the patients were transferred from external hospitals under emergency conditions, resulting in only 24% of patients having a documented medication history. Finally, although we compared 4 surgical strategies, significant baseline heterogeneity (particularly regarding malperfusion status and intimal tear location, both of which represent major determinants of surgical strategy) was still observed, and full adjustment was not feasible.

## CONCLUSION

Total arch replacement using either a CET or an FET demonstrated favourable aortic remodelling in patients with acute type I aortic dissection. The CET may be a reasonable alternative to the FET in terms of aortic remodelling.

## Supplementary Material

ivag023_Supplementary_Data

## Data Availability

The data underlying this article are available from the corresponding author on reasonable request.
