# Supplementary material for: Impact of the Elephant Trunk on Distal Remodelling After Surgery for Acute Type I Aortic Dissection
Source: Interdiscip Cardiovasc Thorac Surg. 2026 Jan 23;41(2):ivag023. doi: 10.1093/icvts/ivag023 (PMC12881956; doi:10.1093/icvts/ivag023)
Supplement: ivag023_Supplementary_Data [file ivag023_supplementary_data.zip › Supplementary_material_20251121_YKP.docx]

**Supplementary Material for the Manuscript**

**Impact of elephant trunk on distal remodelling after surgery for acute type I aortic dissection**

You Kyeong Park1,*, Hyoung Woo Chang2,*, Kay-Hyun Park2, Joon Chul Jung2, Jae Hang Lee2, Jun Sung Kim2

*These two authors contributed equally to this work.

1Department of Thoracic and Cardiovascular Surgery, Soonchunhyang University Bucheon Hospital, Soonchunhyang University College of Medicine, Bucheon, Republic of Korea

2Department of Thoracic and Cardiovascular Surgery, Seoul National University Bundang Hospital, Seoul National University College of Medicine, Seoul, Republic of Korea

**Table of contents**

**Figure S1.** Subgroup analysis of false lumen remodelling: IPTW-weighted KM curves comparing TAR-CET and TAR-FET (A-D)

**Table S1.** Baseline characteristics before and after propensity score matching

|  | Single-source  (n = 425) | Multiple-source (n = 127) | SMD (%) | *P* value | Single-source  (n = 106) | Multiple-source (n = 106) | SMD (%) | *P* value |
| --- | --- | --- | --- | --- | --- | --- | --- | --- |
| Age | 67.1 (11.4) | 67.5 (11.0) | 3.8 | 0.720 | 67.2 (12.6) | 67.3 (10.9) | 0.8 | 0.959 |
| Sex, male | 326 (76.7) | 90 (70.9) | 12.9 | 0.180 | 75 (70.8) | 76 (71.7) | 2.1 | > 0.999 |
| BMI > 30 | 14 (3.3) | 5 (3.9) | 3.3 | 0.782 | 2 (1.9) | 5 (4.7) | 14.6 | 0.453 |
| BMI < 19 | 11 (2.6) | 5 (3.9) | 6.9 | 0.383 | 4 (3.8) | 4 (3.8) | 0.0 | > 0.999 |
| Comorbidity |  |  |  |  |  |  |  |  |
| Hypertension | 316 (74.4) | 89 (70.1) | 9.3 | 0.339 | 81 (76.4) | 75 (70.8) | 12.4 | 0.451 |
| Diabetes mellitus | 274 (64.5) | 71 (55.9) | 17.3 | 0.080 | 62 (58.5) | 63 (59.4) | 1.9 | > 0.999 |
| Hyperlipidemia | 213 (50.1) | 44 (34.6) | 32.5 | 0.002 | 37 (34.9) | 43 (40.6) | 11.9 | 0.451 |
| Chronic kidney disease | 87 (20.5) | 27 (21.3) | 1.9 | 0.847 | 24 (22.6) | 21 (19.8) | 6.9 | 0.711 |
| Cerebrovascular disease | 82 (19.3) | 29 (22.8) | 8.4 | 0.382 | 27 (25.5) | 24 (22.6) | 6.7 | 0.749 |
| Peripheral vascular disease | 89 (20.9) | 19 (15.0) | 16.7 | 0.136 | 12 (11.3) | 17 (16.0) | 13.2 | 0.442 |
| History of PCI | 61 (14.4) | 17 (13.4) | 2.8 | 0.784 | 15 (14.2) | 16 (15.1) | 2.7 | > 0.999 |
| Preoperative status |  |  |  |  |  |  |  |  |
| STEMI | 70 (16.5) | 47 (37.0) | 42.5 | < 0.001 | 32 (30.2) | 33 (31.1) | 2.0 | > 0.999 |
| 3 vessel disease | 381 (89.6) | 115 (90.6) | 3.1 | 0.767 | 96 (90.6) | 96 (90.6) | 0.0 | > 0.999 |
| Left main disease | 158 (37.2) | 45 (35.4) | 3.6 | 0.721 | 36 (34.0) | 39 (36.8) | 5.9 | 0.771 |
| Preoperative ECMO | 6 (1.4) | 7 (5.5) | 18.0 | 0.014 | 5 (4.7) | 3 (2.8) | 8.3 | 0.727 |
| Preoperative IABP | 53 (12.5) | 46 (36.2) | 49.4 | < 0.001 | 29 (27.4) | 28 (26.4) | 2.0 | > 0.999 |
| Symptom onset to surgery (days) | 8.5 (12.4) | 8.6 (18.7) | 0.4 | 0.954 | 8.8 (12.9) | 8.0 (11.4) | 4.4 | 0.619 |
| Culprit lesion PCI | 32 (7.5) | 11 (8.7) | 4.0 | 0.676 | 9 (8.5) | 9 (8.5) | 0.0 | > 0.999 |
| Emergent surgery | 33 (7.8) | 37 (29.1) | 47.0 | < 0.001 | 21 (19.8) | 23 (21.7) | 4.2 | 0.824 |
| Urgent surgery | 324 (76.2) | 73 (57.5) | 37.9 | < 0.001 | 65 (61.3) | 68 (64.2) | 5.7 | 0.761 |
| Isolated CABG | 381 (89.6) | 90 (70.9) | 41.3 | < 0.001 | 86 (81.1) | 80 (75.5) | 12.5 | 0.345 |
| Ejection fraction | 46.1 (14.0) | 39.7 (15.3) | 41.7 | < 0.001 | 42.2 (13.5) | 42.4 (14.7) | 1.7 | 0.870 |

Values are presented as numbers (%) or means (standard deviations).

BMI, body mass index; PCI, percutaneous coronary intervention; STEMI, ST-segment elevation myocardial infarction; ECMO, extracorporeal membrane oxygenation; IABP, intra-aortic balloon pump; CABG, coronary artery bypass grafting; SMD, standardized mean difference.
