# Supplementary material for: Impact of the Elephant Trunk on Distal Remodelling After Surgery for Acute Type I Aortic Dissection
Source: Interdiscip Cardiovasc Thorac Surg. 2026 Jan 23;41(2):ivag023. doi: 10.1093/icvts/ivag023 (PMC12881956; doi:10.1093/icvts/ivag023)
Supplement: ivag023_Supplementary_Data [file ivag023_supplementary_data.zip › FigureS1_IPTW.pdf]

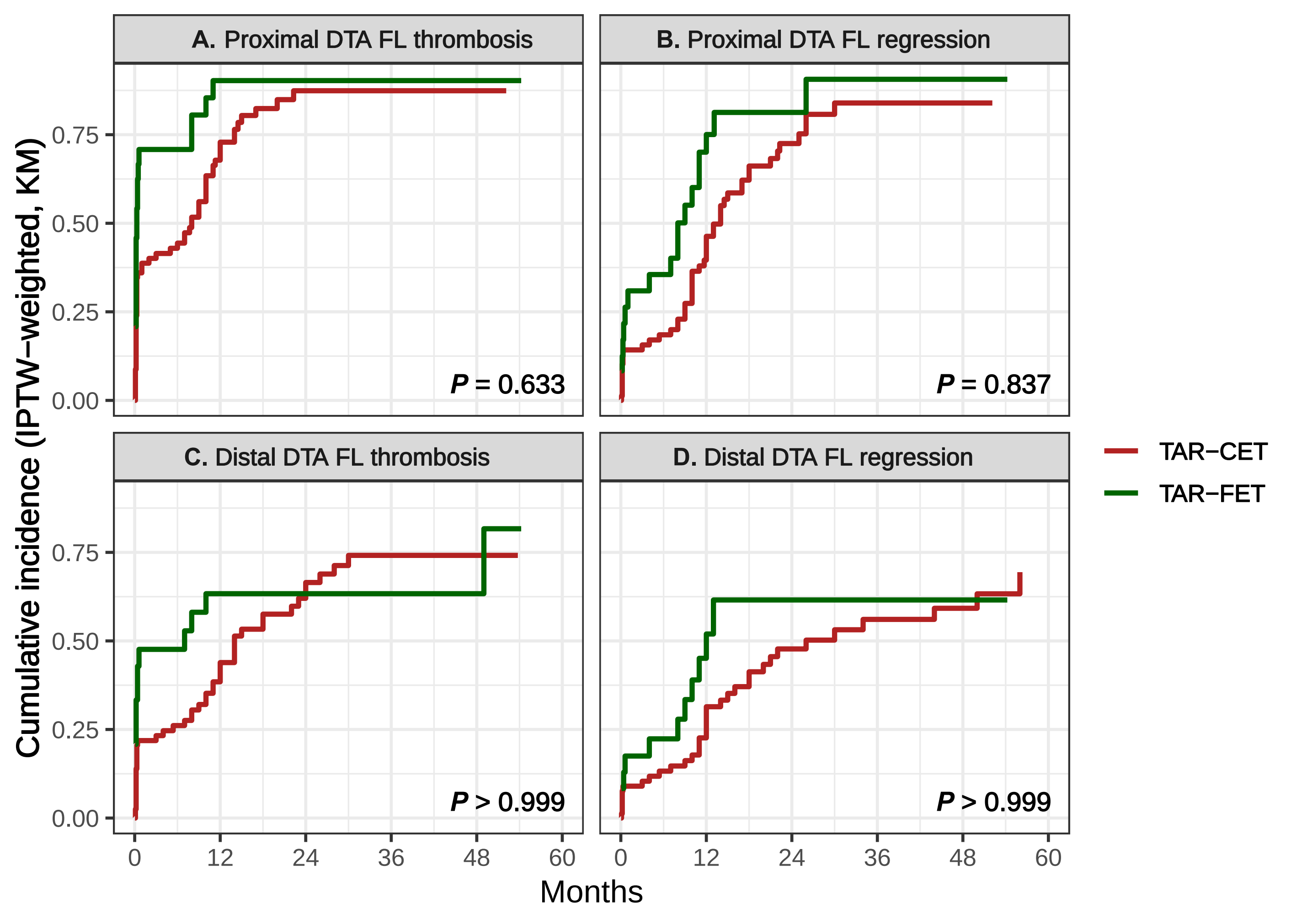
A. Proximal DTA FL thrombosis
B. Proximal DTA FL regression
C. Distal DTA FL thrombosis
D. Distal DTA FL regression
P = 0.633
P = 0.837
P > 0.999
P > 0.999
Cumulative incidence (IPTW−weighted, KM)
0.00
0.25
0.50
0.75
Months
0
12
24
36
48
60
TAR−CET
TAR−FET
