# Supplementary material for: Impact of the Elephant Trunk on Distal Remodelling After Surgery for Acute Type I Aortic Dissection
Source: Interdiscip Cardiovasc Thorac Surg. 2026 Jan 23;41(2):ivag023. doi: 10.1093/icvts/ivag023 (PMC12881956; doi:10.1093/icvts/ivag023)
Supplement: ivag023_Supplementary_Data [file ivag023_supplementary_data.zip › FigureS2_TL_Ao_ratio_final.pdf]

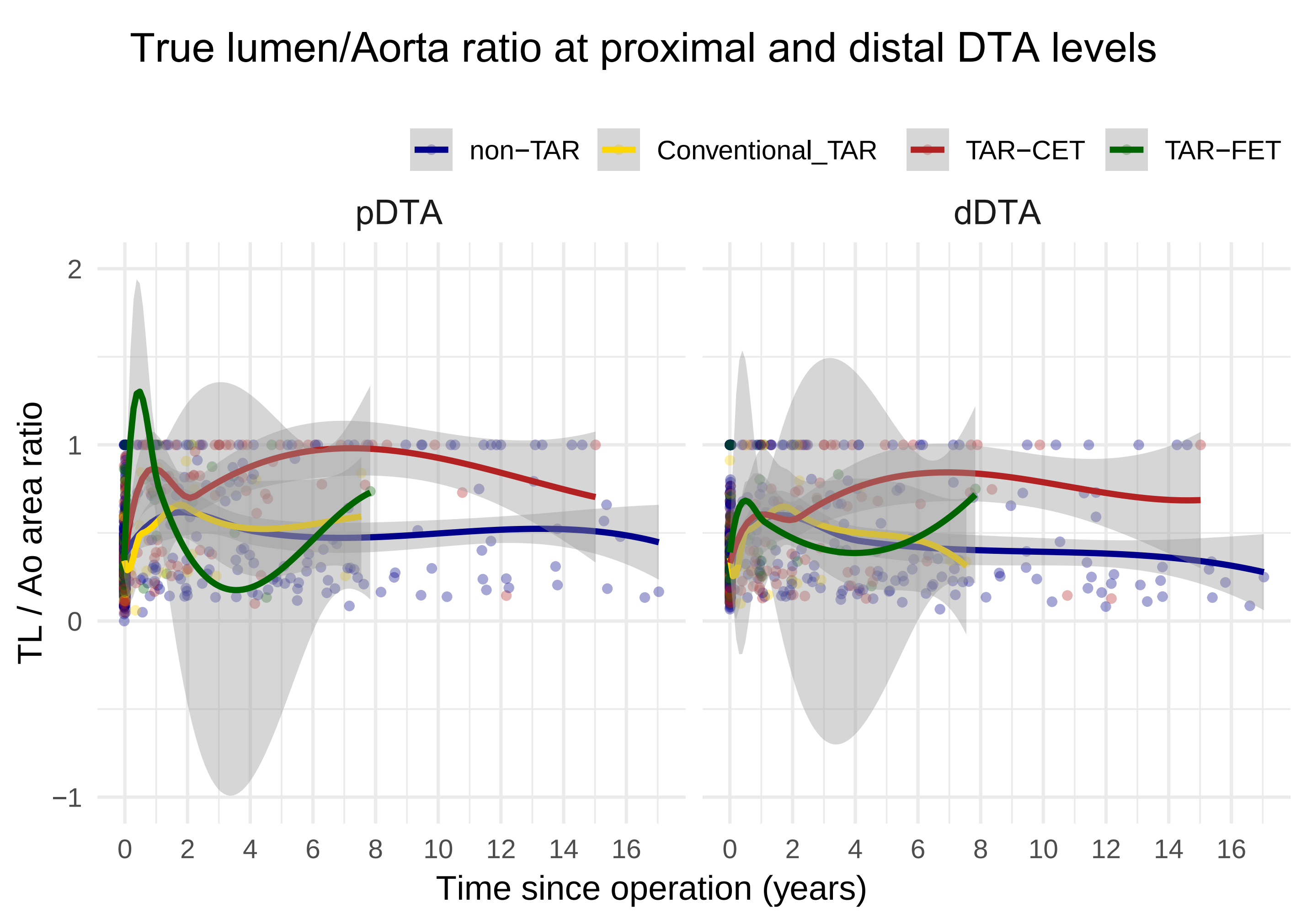
True lumen/Aorta ratio at proximal and distal DTA levels
non−TAR
Conventional_TAR
TAR−CET
TAR−FET
pDTA
dDTA
TL / Ao area ratio
2
1
0
−1
0
2
4
6
8
10
12
14
16
Time since operation (years)
